# Supplementary material for: Inhibition of Sema4D/PlexinB1 signaling alleviates vascular dysfunction in diabetic retinopathy
Source: EMBO Mol Med. 2020 Jan 13;12(2):e10154. doi: 10.15252/emmm.201810154 (PMC7005627; doi:10.15252/emmm.201810154)
Supplement: Supplementary file 1 — Appendix [file EMMM-12-e10154-s001.pdf]

Inhibition of Sema4D/PlexinB1 signaling alleviates vascular dysfunction in diabetic retinopathy

APPENDIX

CONTENTS

Appendix Figure legends

Appendix Figure S1. Sema4D is transcriptionally up-regulated by IRF1

Appendix Figure S2. ADAM17-induced Sema4D shedding in glial cells

Appendix Figure S3. Sema4D deficiency does not influence normal retinal vascular development

Appendix Figure S4. Sema4D/PlexinB1 regulates endothelial cell function depending on phosphorylation of Src and Fak

Appendix Figure S5. Sema4D does not induce pericyte apoptosis but induces pericyte migration

Appendix Figure S6. Sema4D and VEGF have distinct roles in N-cadherin dysfunction of pericytes.

Appendix Figure

Appendix Figure S1

Appendix Figure S2

Appendix Figure S3

Appendix Figure S4

Appendix Figure S5

Appendix Figure S6

Appendix Tables

Appendix Table S1. SiRNA sequence used in this study

Appendix Table S2. Primers used for qPCR

Appendix Table S3. Summary of statistical test and p value

Appendix Figure legends

Appendix Figure S1. Sema4D is transcriptionally up-regulated by IRF1. (A and B) Western blots (A) and quantification (B) showed that the protein levels of IRF1 were up-regulated in primary glial cells after hypoxia in a time-dependent manner (n = 5, *P < 0.05 compared with 0 hour group). (C) The protein levels of IRF1 were silenced by IRF1 siRNA in primary glial cells (n = 5). (D) Silencing IRF1 reversed the increased mRNA levels of Sema4D in primary glial cells after hypoxia (n = 6, *P < 0.05 compared with NT siRNA group, # P < 0.05 compared with NT siRNA+ Hypoxia group). (E and F) Schematic illustration of putative IRF1 binding sequence on the Sema4D promoter region. ChIP image (E) and quantification (F) demonstrated that hypoxia induced the binding of IRF1 to the Sema4D promoter. IgG lane: negative control. (n = 4, *P < 0.05 compared with NT siRNA group, # P < 0.05 compared with NT siRNA+ Hypoxia group). (G and H) Western blots (G) and quantification (H) showed that the IRF1 protein levels were up-regulated in OIR retinas compared with age-matched controls in room air retinas (n = 5, *P < 0.05 compared with room air retinas). (I and J) Western blots (I) and quantification (J) showed that the retinal IRF1 protein levels were up-regulated at six months of diabetes (n = 6, *P < 0.05 compared with vehicle group).

Appendix Figure S2. ADAM17-induced Sema4D shedding in glial cells. (A) Knockdown efficiency for ADAM10, MMP14, ADAMTS4, ADAM17 (n = 3). (B) Silencing ADAM17 but not other metalloproteinases reduced the sSema4D levels in glial cells medium in hypoxia condition (n = 6, *P <

0.05 compared with NT siRNA group). (C) An ADAM17 inhibitor (20 μmol/L TAPI-1) reduced sSema4D concentration in glial cells after hypoxia (n = 6, *P < 0.05 compared with hypoxia alone group). (D and E) Western blots (D) and quantification (E) showed that ADAM17 protein levels in retinas were increased in the OIR model (n = 5, *P < 0.05 compared with room air retinas). (F and G) Western blots (F) and quantification (G) showed that the retinal protein levels of ADAM17 were up-regulated in the STZ model (n = 6, *P < 0.05 compared with vehicle group).

Appendix Figure S3. Sema4D deficiency does not influence normal retinal vascular development. (A) Schematic illustration indicates the target region of the single-guide RNA (sgRNA) in Sema4D gene for Sema4D-knockout (Sema4D-KO) mice. The numbered boxes represent the exons of the gene. The translation starting site (ATG) is in Exon 2 of Sema4D gene, sgRNA target site is highlighted in yellow. (B) Agarose gel image indicated the genomic DNA by PCR analysis of wild-type (WT), Sema4D-KO mice. (C) Western blot analysis confirmed the Sema4D knockout in retinas (n = 6 per group). (D-H) Isolectin B4 staining showed the retinal vasculature at P5 from Sema4D knockout mice and littermate WT controls, the vascular area (E), vascular outgrowth (F), vascular branch points (G) and sprout number (H) were quantified. For Isolectin B4 staining, multiple overlapping (10–20% overlap) images were obtained with a 4 × lens on a fluorescence microscope. The images were merged to visualize the entire retinas. The upper images are representative composite images of entire retinas. The lower images are a cropped enlarged view from the upper entire retina images. (n = 10, bars indicate 100 μm, NS means no statistical significance).

Appendix Figure S4. Sema4D/PlexinB1 regulates endothelial cell function depending on phosphorylation of Src and Fak. (A-E) Endothelial cells pretreated with an inhibitor of Src (KX2-391) were stimulated with or without 1600 ng/ml recombinant Sema4D, and then Western blotting for the phosphorylation of Src, VE-cadherin and Fak after 30 min treatment (A and B), wound-healing (C), TEER value (D) and permeability of dextran (E) were measured (n = 5 in B, C, E. n = 6 in D. *P < 0.05 compared with control group, # P < 0.05 compared with Sema4D group). (F-H) Endothelial cells pretreated with an inhibitor of Fak (GSK2256098) were stimulated with or without 1600 ng/ml recombinant Sema4D, and then wound-healing (F), TEER value (G) and dextran permeability (H) were measured (n = 5 in F, H. n = 6 in G. *P < 0.05 compared with control group, # P < 0.05 compared with Sema4D group).

Appendix Figure S5. Sema4D does not induce pericyte apoptosis but induces pericyte migration.

(A) Representative FACS gating strategy for sorting of endothelial cells and pericytes from retinas. (B) Flow cytometry analysis with anti-CD31 (endothelial cells) or anti-CD13 antibody (pericytes) was used to identify the purity of cultured endothelial cells and pericytes. (C and D) Pericytes were stimulated with different concentrations of recombinant Sema4D for 24 hours and the percentage of apoptosis cells was evaluated by flow cytometry (n = 5, NS means no statistical significance). (E and F) Transwell migration assays showed that Sema4D significantly promoted pericyte migration in a dose-dependent manner (n = 6, bars indicate 100 μm, *P < 0.05 compared with 0 ng/ml Sema4D group).

Appendix Figure S6. Sema4D and VEGF have distinct roles in N-cadherin dysfunction of pericytes. For internalization and immunoprecipitation assays, the cells were treated with recombinant Sema4D or VEGF for four hours. (A and B) Immunoprecipitation and VE-cadherin internalization assays indicated that Sema4D (800 ng/ml) or VEGF (20 ng/ml) displayed a comparable effect on VE-cadherin/p120-catenin/β-catenin dissociation and VE-cadherin internalization in endothelial cells, and both effects could be blocked by a Src inhibitor. (C and D) Immunoprecipitation and N-cadherin internalization assays were performed to measure the effects of Sema4D (800 ng/ml) or VEGF (20 ng/ml) in N-cadherin/p120-catenin/β-catenin dissociation and N-cadherin internalization in pericytes. ($n = 4$ for immunoprecipitation, $n = 6$ for internalization assays, $^*P < 0.05$ compared with control + DMSO group, # $P < 0.05$ compared with corresponding Sema4D + DMSO group or VEGF + DMSO group). (E and F) Endothelial cells (EC) were treated with Sema4D (800 ng/ml) or VEGF (20 ng/ml) for twenty-four hours, Western blotting analyzed the protein levels of VE-cadherin and N-cadherin ($n = 6$, $^*P < 0.05$ compared with control group). (G and H) Pericytes (PC) were treated with Sema4D (800 ng/ml) or VEGF (20 ng/ml) for twenty-four hours, Western blotting analyzed the protein levels of VE-cadherin and N-cadherin ($n = 6$, $^*P < 0.05$ compared with control group). (I) Western blotting compared the protein levels of N-cadherin in endothelial cells and pericytes ($n = 6$). (J and K) Cocultured endothelial cells and pericytes were treated with Sema4D (800 ng/ml) and VEGF (20 ng/ml) for twenty-four hours, Western blotting analyzed the protein levels of VE-cadherin and N-cadherin ($n = 6$, $^*P < 0.05$ compared with control group).

Appendix Figure S1

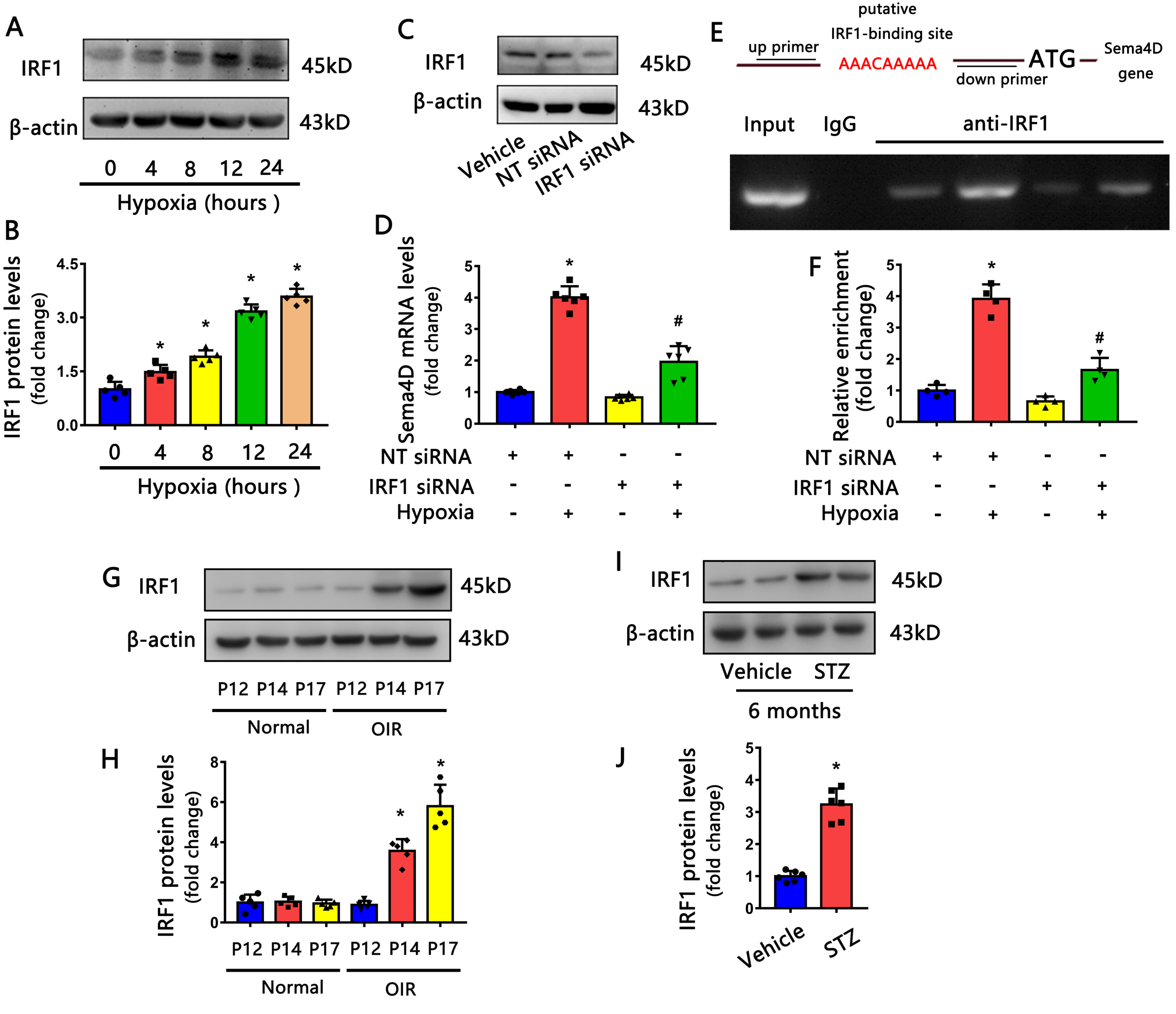

Appendix Figure S2

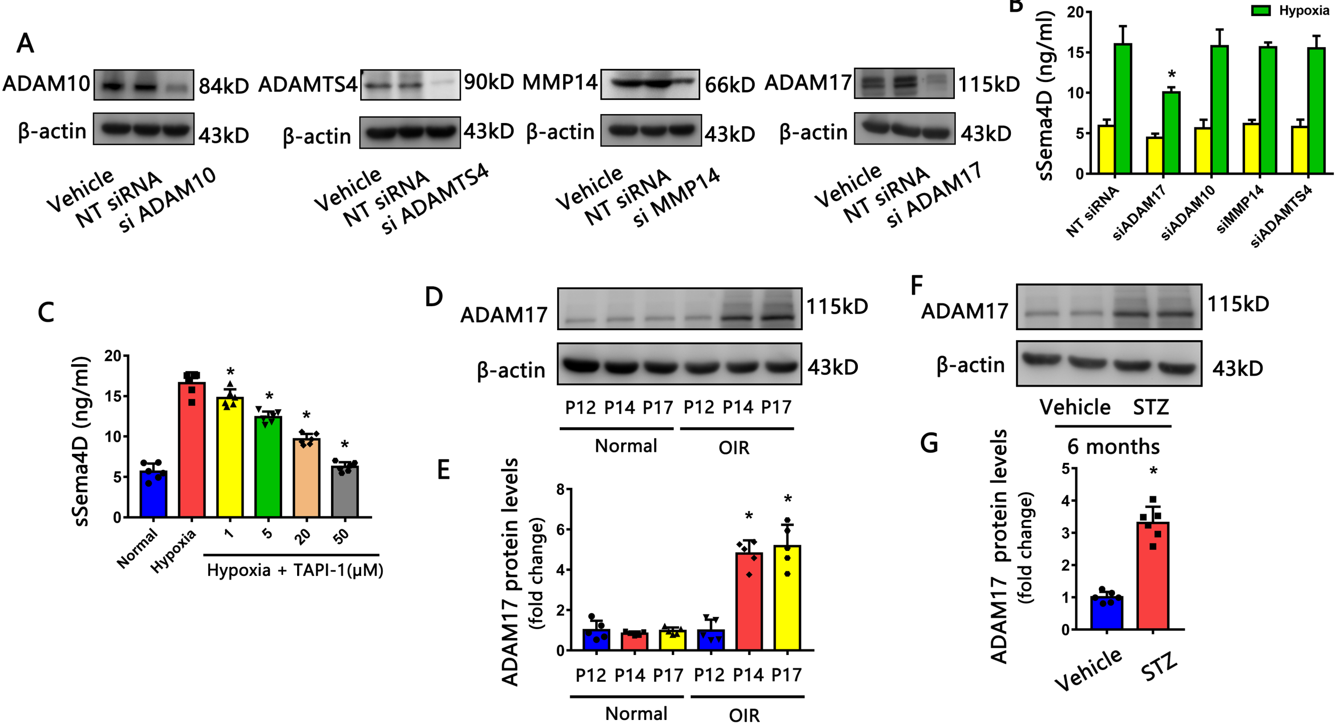

Appendix Figure S3

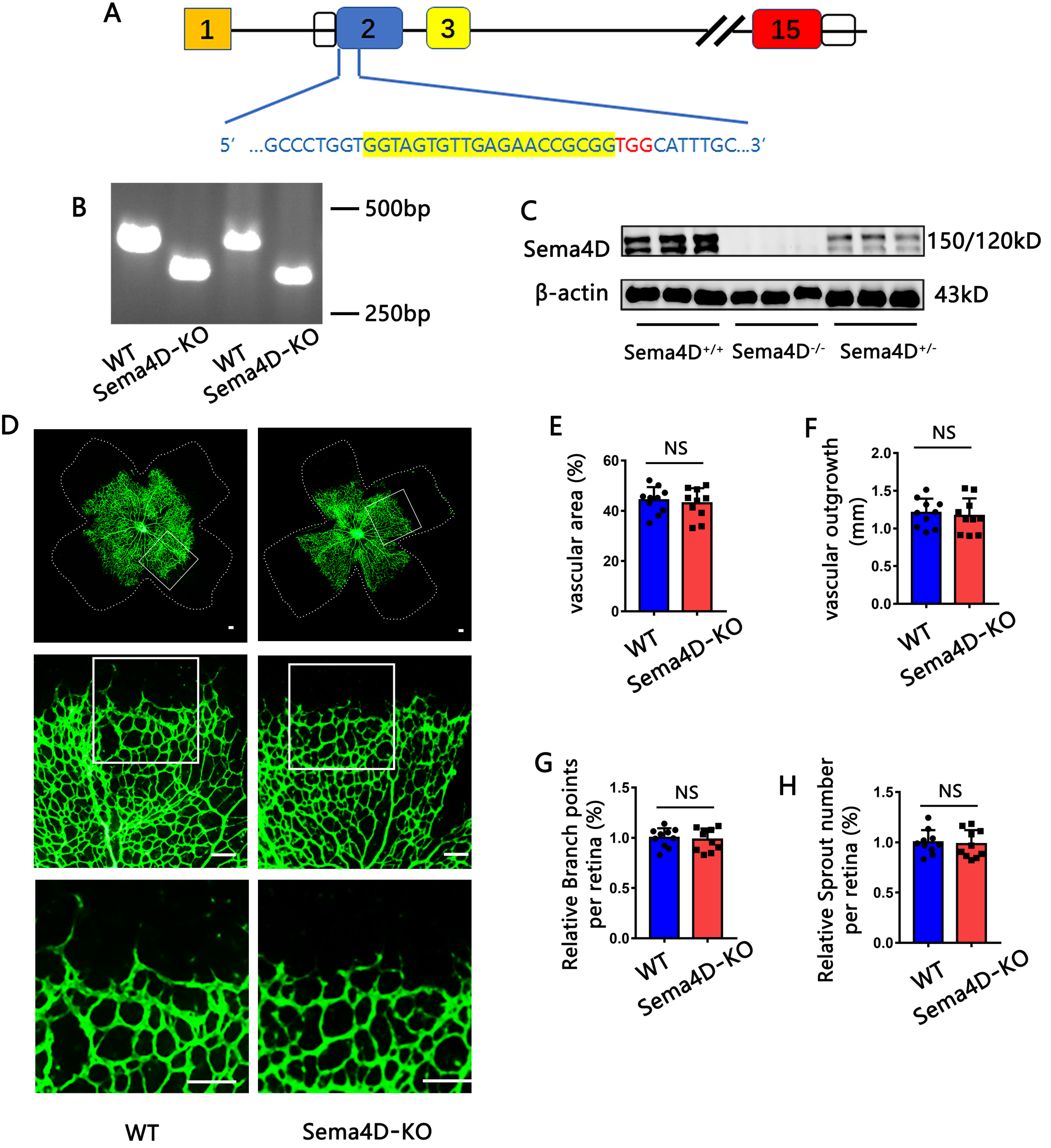

Appendix Figure S4

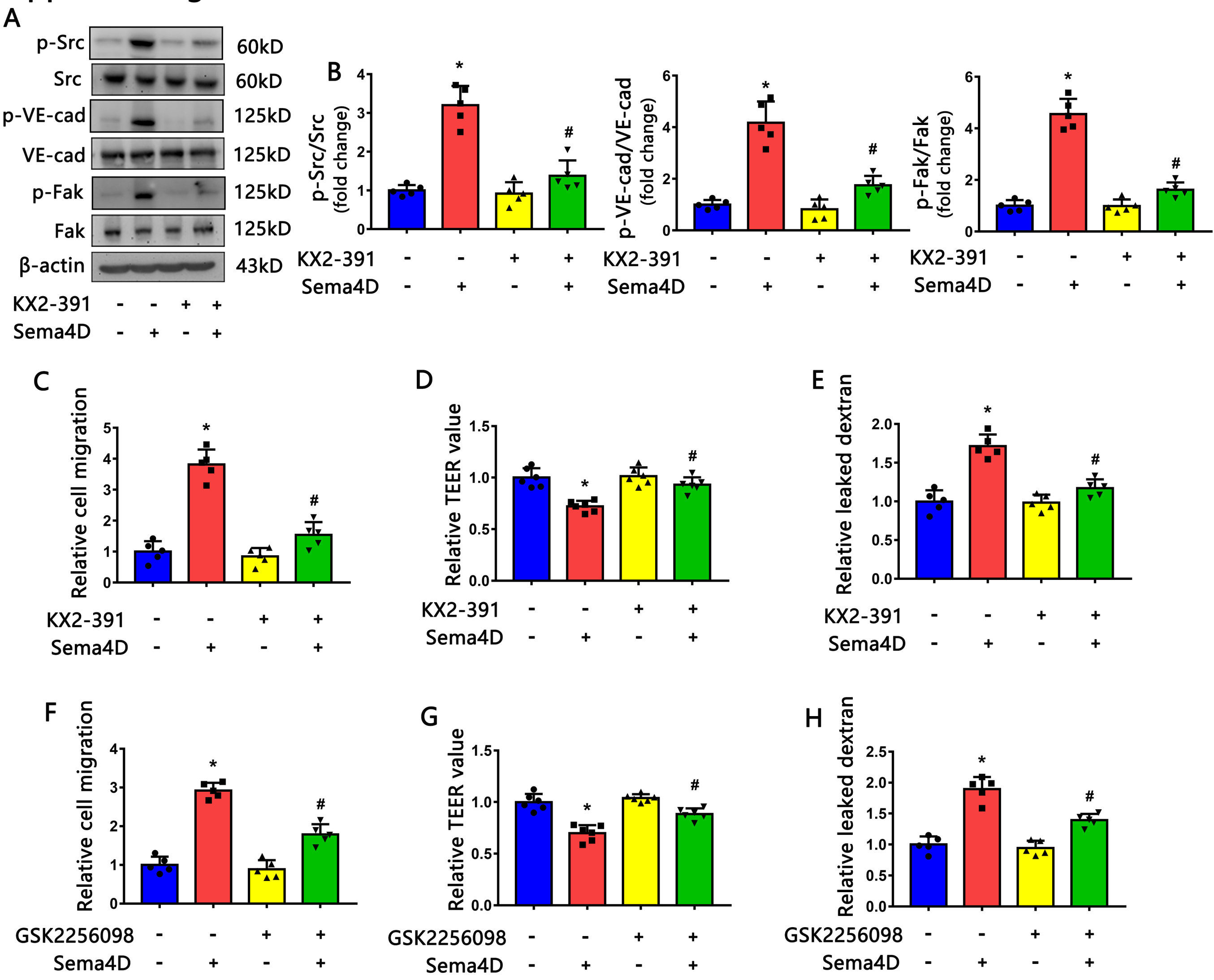

Appendix Figure S5

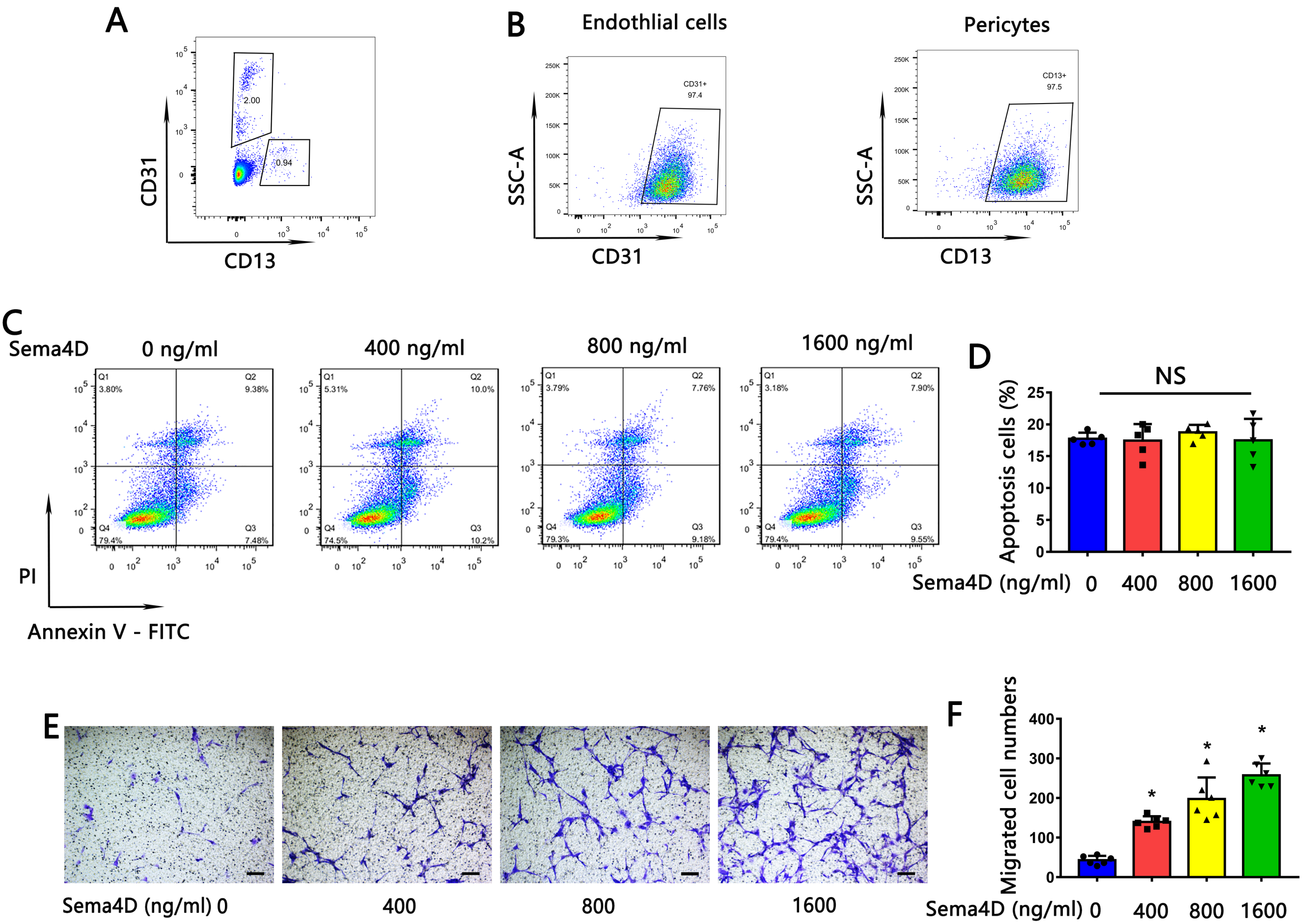

Appendix Figure S6

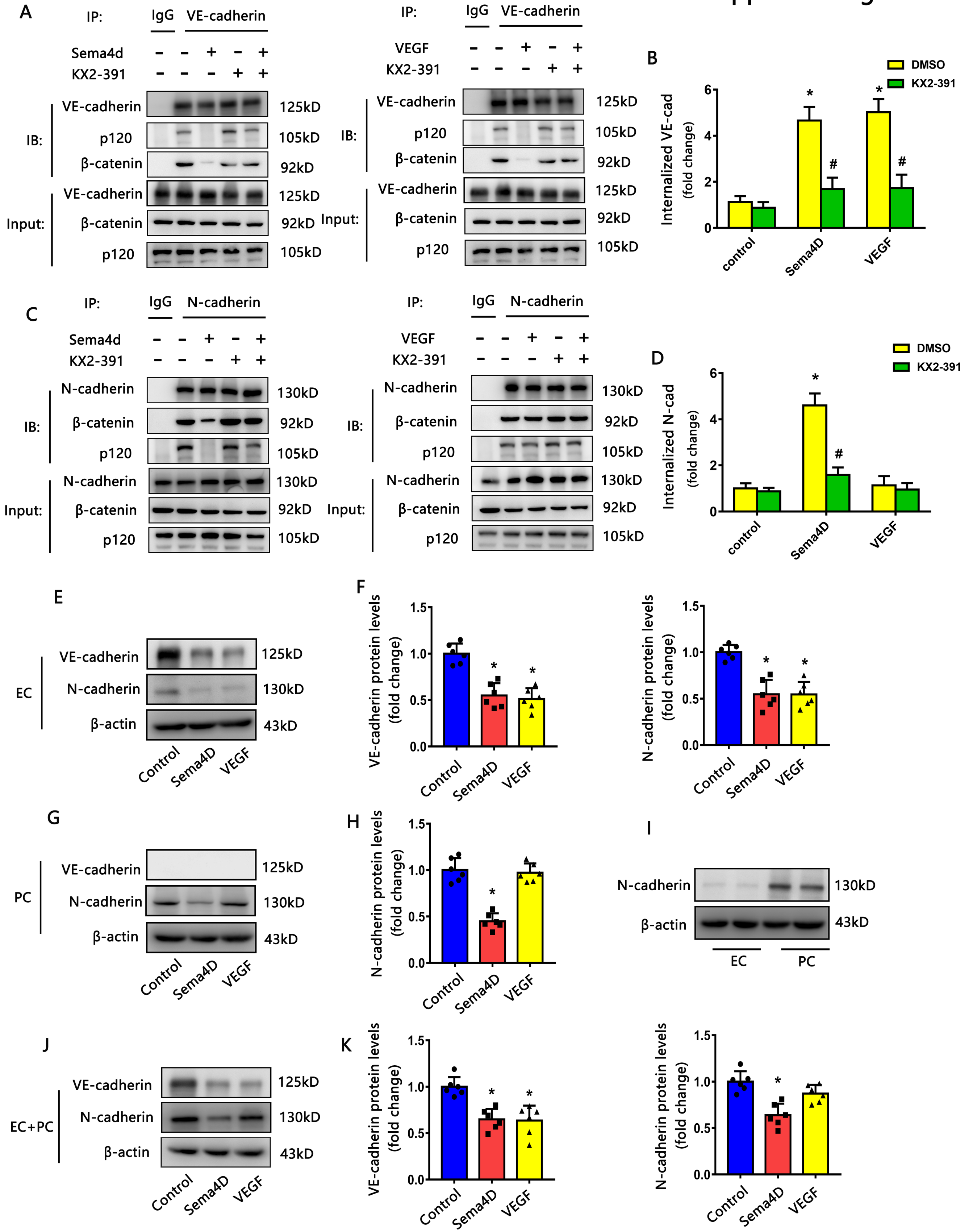

Appendix Table S1. SiRNA sequence used in this study

Gene	SiRNA sequence
IRF1 (mouse)	GGACATTGGGATAGGCATA
ADAM17 (mouse)	CTACAAGACCATAGAAAGT
ADAM10 (mouse)	CCAGTCATGTTAAAGCAAT
MMP14 (mouse)	CGAGAGGAAGGATGGCAAA
ADAMTS4 (mouse)	GGAGATGTTGCTACTAGAA
mDIA1 (mouse)	GCATTTCTTGGCTGAGTTA

Appendix Table S2. Primers used for qPCR

**Gene**	**Forward (5'---3')**	**Reverse (5'---3')**
Sema3A (mouse)	GGCTGGTTCACTGGGATTG	CCGTTTGCATAGTTTGCTCTGG
Sema3B (mouse)	GTAGCAGGGCTAGGGGATACT	AAGGCTTCATAACAGCAGGTC
Sema3C (mouse)	ATGGCATTCCGGGCGATTT	GGTTTTGGTTTCTCGAAGCTCA
Sema3D (mouse)	CTGTATCCCCTTTTTGGGTTCAT	AACCAGACTGAGCAGGAAGAC
Sema3E (mouse)	GGGAGGCAGAGACCTTGTCTA	TGAATGGTGCCCGACTCTGA
Sema3F (mouse)	CATCTGCCTCAACGATGACG	AGAGCCTGAAGAGGTAAAGACA
Sema3G (mouse)	AGGTGGGGAGCTATACACAGG	ACCGGGGTTCATGTAGGAGG
Sema4A (mouse)	ATGGCCCTACCATCCCTGG	AGCAGCGTGTCAAAGTCTCG
Sema4B (mouse)	CAAGACGCTGTATGTGGGGG	TTGACAGTCACGCTTTGGGTC
Sema4C (mouse)	GAGATGTGGTGGAACCTTGTG	CAGGGTCAGTGTCAGGAAGTC
Sema4D (mouse)	CCTGGTGGTAGTGTTGAGAAC	GCAAGGCCGAGTAGTTAAAGAT
Sema4F (mouse)	ATCTCCGAGGCTGACTCCTAT	GCACCGACGTAAAGTGTGTGA
Sema4G (mouse)	TCAGCTTTCTCACAGTAACAGC	CCCTTGAAGTGCCGAATCTGG
Sema5A (mouse)	GCACCCTGTCGTCTCTTACA	TCTTTGTAAGAGACGACAGGGT
Sema5B (mouse)	GAAGCCGTGGGTCTTTAACTT	CAAGAGCAAGCTGGGAGAAAT
Sema6A (mouse)	ACAGCCTGCCCCCTAAAGT	AGCTCCTCTTATATTCGAGCCC
Sema6B (mouse)	GCCCTGTCGTTTTTCCTGCT	ACGGGATAGTGGCTCAAGTAG
Sema6C (mouse)	CTGGACACTGAGGGTCACAG	CGCACGCCATAAGCAGAATC
Sema6D (mouse)	GAGAATCCAATCAGATGGTCCAC	CATGTCACGGTAGCAGTACAC
Sema7A (mouse)	CGTGGCAAGGTCTACCACTT	ACAGACCAGCAGCCCATTAC
PlexinB1 (mouse)	AAGCCCAGCCTACTAACAACC	CAGCCCCACTGTACTGACTG
β-actin (mouse)	CCAGTTGGTAACAATGCCATGT	GGCTGTATTCCCCTCCATCG

Appendix Table S3. Summary of statistical test and p value

Figure number	Test used	Sample	P value
Fig 1A	unpaired two-tailed Student's t-test	Sema4D	<0.0001
		Sema3G	<0.0001
		Sema5B	0.0028
		Sema3A	0.0150
		Sema3F	0.0272
Fig 1B	unpaired two-tailed Student's t-test	Sema4D	<0.0001
		Sema4F	<0.0001
		Sema5B	0.0004
		Sema6B	0.0006
		Sema3A	0.0008
		Sema4A	0.0034
		Sema3D	0.0044
		Sema3E	0.0052
		Sema3C	0.0180
Fig 1D	Mann Whitney test	DR vs. Control	<0.0001
Fig 1E	Spearman's rank test		<0.0001
Fig 1F	Spearman's rank test		0.0002
Fig 2A	One-way ANOVA followed by a Tukey's post hoc test	Normal P14 vs. OIR P14	<0.0001
		Normal P17 vs. OIR P17	<0.0001
Fig 2C	One-way ANOVA followed by a Tukey's post hoc test	Normal P14 vs. OIR P14	<0.0001
		Normal P17 vs. OIR P17	<0.0001
Fig 2E	unpaired two-tailed Student's t-test		<0.0001
Fig 2G	unpaired two-tailed Student's t-test		<0.0001
Fig 2J	One-way ANOVA followed by a Tukey's post hoc test	0 hour vs. 4 hours	0.0188
		0 hour vs. 8 hours	0.0004
		0 hour vs. 12 hours	0.0019
		0 hour vs. 24 hours	0.0018
Fig 2K	One-way ANOVA followed by a Tukey's post hoc test	0 hour vs. 4 hours	0.0032
		0 hour vs. 8 hours	<0.0001
		0 hour vs. 12 hours	<0.0001
		0 hour vs. 24 hours	<0.0001
Fig 3B	unpaired two-tailed Student's t-test		<0.0001
Fig 3C	unpaired two-tailed Student's t-test		<0.0001

Fig 3E	unpaired two-tailed Student's t-test		<0.0001
Fig 3G	One-way ANOVA followed by a Tukey's post hoc test	WT + Vehicle vs. WT + STZ	<0.0001
		WT + STZ vs. Sema4D-KO+STZ	<0.0001
Fig 3I	One-way ANOVA followed by a Tukey's post hoc test	WT + Vehicle vs. WT + STZ	<0.0001
		WT + STZ vs. Sema4D-KO+STZ	<0.0001
Fig 3K	One-way ANOVA followed by a Tukey's post hoc test	WT + Vehicle vs. WT + STZ	<0.0001
		WT + STZ vs. Sema4D-KO+STZ	0.0012
Fig 3L	One-way ANOVA followed by a Tukey's post hoc test	WT + Vehicle vs. WT + STZ	<0.0001
		WT + STZ vs. Sema4D-KO+STZ	0.0007
Fig 4C	One-way ANOVA followed by a Tukey's post hoc test	5 min vs. 0 min for p-Src	0.0002
		15 min vs. 0 min for p-Src	<0.0001
		30 min vs. 0 min for p-Src	<0.0001
		60 min vs. 0 min for p-Src	0.0024
		15 min vs. 0 min for p-VE-cadherin	<0.0001
		30 min vs. 0 min for p-VE-cadherin	<0.0001
		60 min vs. 0 min for p-VE-cadherin	0.0082
		5 min vs. 0 min for p-Fak	0.0465
		15 min vs. 0 min for p-Fak	<0.0001
		30 min vs. 0 min for p-Fak	0.0001
		60 min vs. 0 min for p-Fak	0.0108
Fig 4D	One-way ANOVA followed by a Tukey's post hoc test	mDIA1 siRNA vs. NT siRNA	<0.0001
Fig 4F	One-way ANOVA followed by a Tukey's post hoc test	Sema4D+NT siRNA vs. NT siRNA for p-Src	<0.0001
		Sema4D+mDIA1 siRNA vs. Sema4D+NT siRNA for p-Src	<0.0001
		Sema4D+NT siRNA vs. NT siRNA for p-VE-cadherin	<0.0001
		Sema4D+mDIA1 siRNA vs. Sema4D +NT siRNA for p-VE-cadherin	0.0002
		Sema4D+NT siRNA vs. NT siRNA for p-Fak	<0.0001
		Sema4D+ mDIA1 siRNA vs. Sema4D+NT siRNA for p-Fak	<0.0001
Fig 4H	One-way ANOVA followed by a Tukey's post hoc test	Sema4D+NT siRNA vs. NT siRNA	<0.0001
		Sema4D+mDIA1 siRNA vs. Sema4D+NT siRNA	<0.0001

Fig 4I	One-way ANOVA followed by a Tukey's post hoc test	Sema4D+NT siRNA vs. NT siRNA	<0.0001
		Sema4D+mDIA1 siRNA vs. Sema4D+NT siRNA	0.0017
Fig 4J	One-way ANOVA followed by a Tukey's post hoc test	Sema4D+NT siRNA vs. NT siRNA	<0.0001
		Sema4D+mDIA1 siRNA vs. Sema4D+NT siRNA	0.0002
Fig 4L	One-way ANOVA followed by a Tukey's post hoc test	Sema4D+NT siRNA vs. NT siRNA	<0.0001
		Sema4D+mDIA1 siRNA vs. Sema4D+NT siRNA	<0.0001
Fig 5A	One-way ANOVA followed by a Tukey's post hoc test	400 vs. 0 ng/ml Sema4D	0.0005
		800 vs. 0 ng/ml Sema4D	<0.0001
		1600 vs. 0 ng/ml Sema4D	<0.0001
Fig 5B	One-way ANOVA followed by a Tukey's post hoc test	400 vs. 0 ng/ml Sema4D	0.0078
		800 vs. 0 ng/ml Sema4D	<0.0001
		1600 vs. 0 ng/ml Sema4D	<0.0001
Fig 5C	unpaired two-tailed Student's t-test		<0.0001
Fig 5E	unpaired two-tailed Student's t-test		<0.0001
Fig 5G	One-way ANOVA followed by a Tukey's post hoc test	CRISPR-plB1 vs. CRISPR-wt	<0.0001
Fig 5H	Two-way ANOVA followed by a Tukey's post hoc test	PC-CRISPR-plB1+Sema4D vs. CRISPR-wt+Sema4D	<0.0001
		EC-CRISPR-plB1+Sema4D vs. CRISPR-wt+Sema4D	<0.0001
Fig 5I	Two-way ANOVA followed by a Tukey's post hoc test	PC-CRISPR-plB1+Sema4D vs. CRISPR-wt+Sema4D	<0.0001
		EC-CRISPR-plB1+Sema4D vs. CRISPR-wt+Sema4D	<0.0001
Fig 5K	One-way ANOVA followed by a Tukey's post hoc test	CRISPR-wt+Sema4D vs. CRISPR-wt	<0.0001
		CRISPR-wt+Sema4D vs. CRISPR-plB1+Sema4D	<0.0001
Fig 5M	One-way ANOVA followed by a Tukey's post hoc test	5 min vs. 0 min for p-Src	0.0040
		15 min vs. 0 min for p-Src	<0.0001
		30 min vs. 0 min for p-Src	<0.0001
		60 min vs. 0 min for p-Src	0.0001
Fig 5P	One-way ANOVA followed by a Tukey's post hoc test	Sema4D vs. Control	<0.0001

		Sema4D vs. KX2-391+Sema4D	<0.0001
Fig 5S	One-way ANOVA followed by a Tukey's post hoc test	Sema4D vs. Control	<0.0001
		Sema4D vs. KX2-391+Sema4D	<0.0001
Fig 6D	One-way ANOVA followed by a Tukey's post hoc test	shRNA-plB1 vs. NT shRNA group	0.0011
Fig 6F	unpaired two-tailed Student's t-test	shRNA-plB1 vs. NT shRNA group	0.0005
Fig 6G	unpaired two-tailed Student's t-test	shRNA-plB1 vs. NT shRNA group	0.0007
Fig 6K	One-way ANOVA followed by a Tukey's post hoc test	Tie2-Cre + shRNA-plB1 vs. Tie2-Cre + NT shRNA group	0.0003
		PDGFRβ-Cre + shRNA-plB1 vs. PDGFRβ-Cre + NT shRNA group	0.0006
Fig 6M	One-way ANOVA followed by a Tukey's post hoc test	Tie2-Cre + shRNA-plB1 vs. Tie2-Cre + NT shRNA group	0.0001
		PDGFRβ-Cre + shRNA-plB1 vs. PDGFRβ-Cre + NT shRNA group	<0.0001
Fig 7A	One-way ANOVA followed by a Tukey's post hoc test	0.5 μg anti-Sema4D vs. IgG	0.0235
		1 μg anti-Sema4D vs. IgG	<0.0001
		2 μg anti-Sema4D vs. IgG	<0.0001
Fig 7B	One-way ANOVA followed by a Tukey's post hoc test	0.5 μg anti-Sema4D vs. IgG	0.0002
		1 μg anti-Sema4D vs. IgG	<0.0001
		2 μg anti-Sema4D vs. IgG	<0.0001
Fig 7C	One-way ANOVA followed by a Tukey's post hoc test	WT+ anti-Sema4D vs. WT+ IgG	<0.0001
		Sema4D-KO + anti-Sema4D vs. Sema4D-KO + IgG	0.9915
Fig 7D	One-way ANOVA followed by a Tukey's post hoc test	WT+ anti-Sema4D vs. WT+ IgG	<0.0001
		Sema4D-KO + anti-Sema4D vs. Sema4D-KO + IgG	0.8557
Fig 7F	One-way ANOVA followed by a Tukey's post hoc test	anti-Sema4D vs. IgG	<0.0001
		anti-VEGF vs. IgG	<0.0001
		anti-VEGF vs. anti- Sema4D	0.3745
		anti-VEGF + anti-Sema4D vs. anti-VEGF	<0.0001
Fig 7G	One-way ANOVA followed by a Tukey's post hoc test	anti-Sema4D vs. IgG	<0.0001
		anti-VEGF vs. IgG	<0.0001
		anti-VEGF vs. anti- Sema4D	0.2383
		anti-VEGF + anti-Sema4D vs.	0.0005

		anti-VEGF	
Fig 7I	One-way ANOVA followed by a Tukey's post hoc test	anti-Sema4D vs. IgG	<0.0001
		anti-VEGF vs. IgG	<0.0001
		anti-VEGF vs. anti- Sema4D	0.7431
		anti-VEGF + anti-Sema4D vs. anti-VEGF	0.0080
Fig 7K	One-way ANOVA followed by a Tukey's post hoc test	anti-Sema4D vs. IgG for p-Src	<0.0001
		anti-VEGF vs. IgG for p-Src	<0.0001
		anti-VEGF vs. anti- Sema4D for p-Src	0.7409
		anti-VEGF + anti-Sema4D vs. anti-VEGF for p-Src	0.0045
	One-way ANOVA followed by a Tukey's post hoc test	anti-Sema4D vs. IgG for p-VE-cad	<0.0001
		anti-VEGF vs. IgG for p-VE-cad	<0.0001
		anti-VEGF vs. anti- Sema4D for p-VE-cad	0.9819
		anti-VEGF + anti-Sema4D vs. anti-VEGF for p-VE-cad	0.0121
	One-way ANOVA followed by a Tukey's post hoc test	anti-Sema4D vs. IgG for p-Fak	<0.0001
		anti-VEGF vs. IgG for p-Fak	<0.0001
		anti-VEGF vs. anti- Sema4D for p-Fak	0.9412
		anti-VEGF + anti-Sema4D vs. anti-VEGF for p-Fak	0.0228
Fig 7M	One-way ANOVA followed by a Tukey's post hoc test	anti-Sema4D vs. IgG	0.0006
		anti-VEGF vs. IgG	<0.0001
		anti-VEGF vs. anti- Sema4D	0.6974
		anti-VEGF + anti-Sema4D vs. anti-VEGF	0.0064
Fig 8B	One-way ANOVA followed by a Tukey's post hoc test	anti-Sema4D vs. IgG	<0.0001
		anti-VEGF vs. IgG	<0.0001
		anti-VEGF vs. anti- Sema4D	0.0627
		anti-VEGF + anti-Sema4D vs. anti-Sema4D	<0.0001
Fig 8D	One-way ANOVA followed by a Tukey's post hoc test	anti-Sema4D vs. IgG	<0.0001
		anti-VEGF vs. IgG	0.0086

		anti-VEGF vs. anti- Sema4D	0.0180
		anti-VEGF + anti-Sema4D vs. anti-Sema4D	0.0408
Fig 8E	One-way ANOVA followed by a Tukey's post hoc test	anti-Sema4D vs. IgG	<0.0001
		anti-VEGF vs. IgG	0.0108
		anti-VEGF vs. anti- Sema4D	0.0004
		anti-VEGF + anti-Sema4D vs. anti-Sema4D	0.0213
Fig 8F	One-way ANOVA followed by a Tukey's post hoc test	anti-Sema4D vs. IgG	<0.0001
		anti-VEGF vs. IgG	0.0052
		anti-VEGF vs. anti- Sema4D	0.0002
		anti-VEGF + anti-Sema4D vs. anti-Sema4D	0.0119
Fig 8H	One-way ANOVA followed by a Tukey's post hoc test	anti-Sema4D vs. IgG	<0.0001
		anti-VEGF vs. IgG	<0.0001
		anti-VEGF vs. anti- Sema4D	0.9947
		anti-VEGF + anti-Sema4D vs. anti-Sema4D	<0.0001
Fig 8J	One-way ANOVA followed by a Tukey's post hoc test	anti-Sema4D vs. IgG	<0.0001
		anti-VEGF vs. IgG	0.2081
		anti-VEGF vs. anti- Sema4D	0.0222
		anti-VEGF + anti-Sema4D vs. anti-Sema4D	0.6156
Fig 8L	One-way ANOVA followed by a Tukey's post hoc test	anti-Sema4D vs. IgG	<0.0001
		anti-VEGF vs. IgG	<0.0001
		anti-VEGF vs. anti- Sema4D	<0.0001
		anti-VEGF + anti-Sema4D vs. anti-Sema4D	<0.0001
Fig EV1B	One-way ANOVA followed by a Tukey's post hoc test	WT + Vehicle vs. WT + STZ	<0.0001
		WT + STZ vs. Sema4D-KO+STZ	0.0083
Fig EV1D	One-way ANOVA followed by a Tukey's post hoc test	WT + Vehicle vs. WT + STZ	<0.0001
		WT + STZ vs. Sema4D-KO+STZ	<0.0001
Fig EV2B	One-way ANOVA followed by a Tukey's post hoc test	400 vs. 0 ng/ml Sema4D	0.3227
		800 vs. 0 ng/ml Sema4D	<0.0001

		1600 vs. 0 ng/ml Sema4D	<0.0001
Fig EV2C	One-way ANOVA followed by a Tukey's post hoc test	400 vs. 0 ng/ml Sema4D	0.0247
		800 vs. 0 ng/ml Sema4D	0.0003
		1600 vs. 0 ng/ml Sema4D	<0.0001
Fig EV2D	One-way ANOVA followed by a Tukey's post hoc test	400 vs. 0 ng/ml Sema4D	0.0201
		800 vs. 0 ng/ml Sema4D	0.0002
		1600 vs. 0 ng/ml Sema4D	<0.0001
Fig EV2E	One-way ANOVA followed by a Tukey's post hoc test	CRISPR-plB1 vs. CRISPR-wt	<0.0001
Fig EV2G	One-way ANOVA followed by a Tukey's post hoc test	CRISPR-wt+Sema4D vs. CRISPR-wt	<0.0001
		CRISPR-wt+Sema4D vs. CRISPR-plB1+Sema4D	<0.0001
Fig EV2H	One-way ANOVA followed by a Tukey's post hoc test	CRISPR-wt+Sema4D vs. CRISPR-wt	<0.0001
		CRISPR-wt+Sema4D vs. CRISPR-plB1+Sema4D	0.0004
Fig EV2I	One-way ANOVA followed by a Tukey's post hoc test	CRISPR-wt+Sema4D vs. CRISPR-wt	<0.0001
		CRISPR-wt+Sema4D vs. CRISPR-plB1+Sema4D	<0.0001
Fig EV3B	One-way ANOVA followed by a Tukey's post hoc test	WT + Normal vs. WT + OIR for p-Src	<0.0001
		WT + OIR vs. Sema4D-KO+OIR for p-Src	0.0350
		WT + Normal vs. WT + OIR for p-VE-cadherin	<0.0001
		WT + OIR vs. Sema4D-KO+OIR for p-VE-cadherin	0.0306
		WT + Normal vs. WT + OIR for p-Fak	<0.0001
		WT + OIR vs. Sema4D-KO+OIR for p-Fak	0.0125
Fig EV3D	One-way ANOVA followed by a Tukey's post hoc test	WT + Vehicle vs. WT + STZ	<0.0001
		WT + STZ vs. Sema4D-KO+STZ	<0.0001
Fig EV3F	One-way ANOVA followed by a Tukey's post hoc test	WT + Vehicle vs. WT + STZ	<0.0001
		WT + STZ vs. Sema4D-KO+STZ	<0.0001
Fig S1B	One-way ANOVA followed by a Tukey's post hoc test	0 hour vs. 4 hours	0.0095
		0 hour vs. 8 hours	<0.0001

		0 hour vs. 12 hours	<0.0001
		0 hour vs. 24 hours	<0.0001
Fig S1D	One-way ANOVA followed by a Tukey's post hoc test	Hypoxia + NT siRNA vs. NT siRNA	<0.0001
		Hypoxia + NT siRNA vs. Hypoxia + IRF1 siRNA	<0.0001
Fig S1F	One-way ANOVA followed by a Tukey's post hoc test	Hypoxia + NT siRNA vs. NT siRNA	<0.0001
		Hypoxia + NT siRNA vs. Hypoxia + IRF1 siRNA	<0.0001
Fig S1H	One-way ANOVA followed by a Tukey's post hoc test	Normal P14 vs. OIR P14	<0.0001
		Normal P17 vs. OIR P17	<0.0001
Fig S1J	unpaired two-tailed Student's t-test		<0.0001
Fig S2B	Two-way ANOVA followed by a Tukey's post hoc test	Hypoxia + NT siRNA vs. Hypoxia + ADAM17 siRNA	<0.0001
Fig S2C	One-way ANOVA followed by a Tukey's post hoc test	Hypoxia vs. 1	0.0234
		Hypoxia vs. 5	<0.0001
		Hypoxia vs. 20	<0.0001
		Hypoxia vs. 50	<0.0001
Fig S2E	One-way ANOVA followed by a Tukey's post hoc test	Normal P14 vs. OIR P14	<0.0001
		Normal P17 vs. OIR P17	<0.0001
Fig S2G	unpaired two-tailed Student's t-test		<0.0001
Fig S3E	unpaired two-tailed Student's t-test		0.6246
Fig S3F	unpaired two-tailed Student's t-test		0.6817
Fig S3G	unpaired two-tailed Student's t-test		0.6915
Fig S3H	unpaired two-tailed Student's t-test		0.7666
Fig S4B	One-way ANOVA followed by a Tukey's post hoc test	Sema4D vs. Control for p-Src	<0.0001
		Sema4D+KX2-391 vs. Sema4D for p-Src	<0.0001
		Sema4D vs. Control for p-VE-cadherin	<0.0001
		Sema4D+KX2-391 vs. Sema4D for p-VE-cadherin	<0.0001
		Sema4D vs. Control for p-Fak	<0.0001
		Sema4D+KX2-391 vs. Sema4D for p-p-Fak	<0.0001
Fig S4C	One-way ANOVA followed by a Tukey's post hoc test	Sema4D vs. Control	<0.0001
		Sema4D+KX2-391 vs. Sema4D	<0.0001

Fig S4D	One-way ANOVA followed by a Tukey's post hoc test	Sema4D vs. Control	<0.0001
		Sema4D+KX2-391 vs. Sema4D	0.0008
Fig S4E	One-way ANOVA followed by a Tukey's post hoc test	Sema4D vs. Control	<0.0001
		Sema4D+KX2-391 vs. Sema4D	<0.0001
Fig S4F	One-way ANOVA followed by a Tukey's post hoc test	Sema4D vs. Control	<0.0001
		Sema4D+GSK2256098 vs. Sema4D	<0.0001
Fig S4G	One-way ANOVA followed by a Tukey's post hoc test	Sema4D vs. Control	<0.0001
		Sema4D+GSK2256098 vs. Sema4D	0.0004
Fig S4H	One-way ANOVA followed by a Tukey's post hoc test	Sema4D vs. Control	<0.0001
		Sema4D+GSK2256098 vs. Sema4D	0.0002
Fig S5D	One-way ANOVA followed by a Tukey's post hoc test	400 vs. 0 ng/ml Sema4D	0.9972
		800 vs. 0 ng/ml Sema4D	0.8956
		1600 vs. 0 ng/ml Sema4D	0.9982
Fig S5F	One-way ANOVA followed by a Tukey's post hoc test	400 vs. 0 ng/ml Sema4D	0.0003
		800 vs. 0 ng/ml Sema4D	<0.0001
		1600 vs. 0 ng/ml Sema4D	<0.0001
Fig S6B	Two-way ANOVA followed by a Tukey's post hoc test	Sema4D+DMSO vs. control +DMSO	<0.0001
		VEGF+DMSO vs. control +DMSO	<0.0001
		Sema4D+DMSO vs. Sema4D + KX2-391	<0.0001
		VEGF+DMSO vs. VEGF + KX2-391	<0.0001
Fig S6D	Two-way ANOVA followed by a Tukey's post hoc test	Sema4D+DMSO vs. control +DMSO	<0.0001
		VEGF+DMSO vs. control +DMSO	0.9802
		Sema4D+DMSO vs. Sema4D + KX2-391	<0.0001
		VEGF+DMSO vs. VEGF + KX2-391	0.9353
Fig S6F	One-way ANOVA followed by a Tukey's post hoc test	Sema4D vs. Control for VE-cadherin	<0.0001
		VEGF vs. Control for VE-cadherin	<0.0001
		Sema4D vs. Control for N-cadherin	<0.0001
		VEGF vs. Control for N-cadherin	<0.0001
Fig S6H	One-way ANOVA followed by a Tukey's post hoc test	Sema4D vs. Control for N-cadherin	<0.0001
		VEGF vs. Control for N-cadherin	0.8904

Fig S6K	One-way ANOVA followed by a Tukey's post hoc test	Sema4D vs. Control for VE-cadherin	0.0007
		VEGF vs. Control for VE-cadherin	0.0005
		Sema4D vs. Control for N-cadherin	0.0001
		VEGF vs. Control for N-cadherin	0.1465
