# Supplementary material for: Inhibition of Sema4D/PlexinB1 signaling alleviates vascular dysfunction in diabetic retinopathy
Source: EMBO Mol Med. 2020 Jan 13;12(2):e10154. doi: 10.15252/emmm.201810154 (PMC7005627; doi:10.15252/emmm.201810154)
Supplement: Supplementary file 2 — Expanded View Figures PDF [file EMMM-12-e10154-s002.pdf]

Expanded View Figures

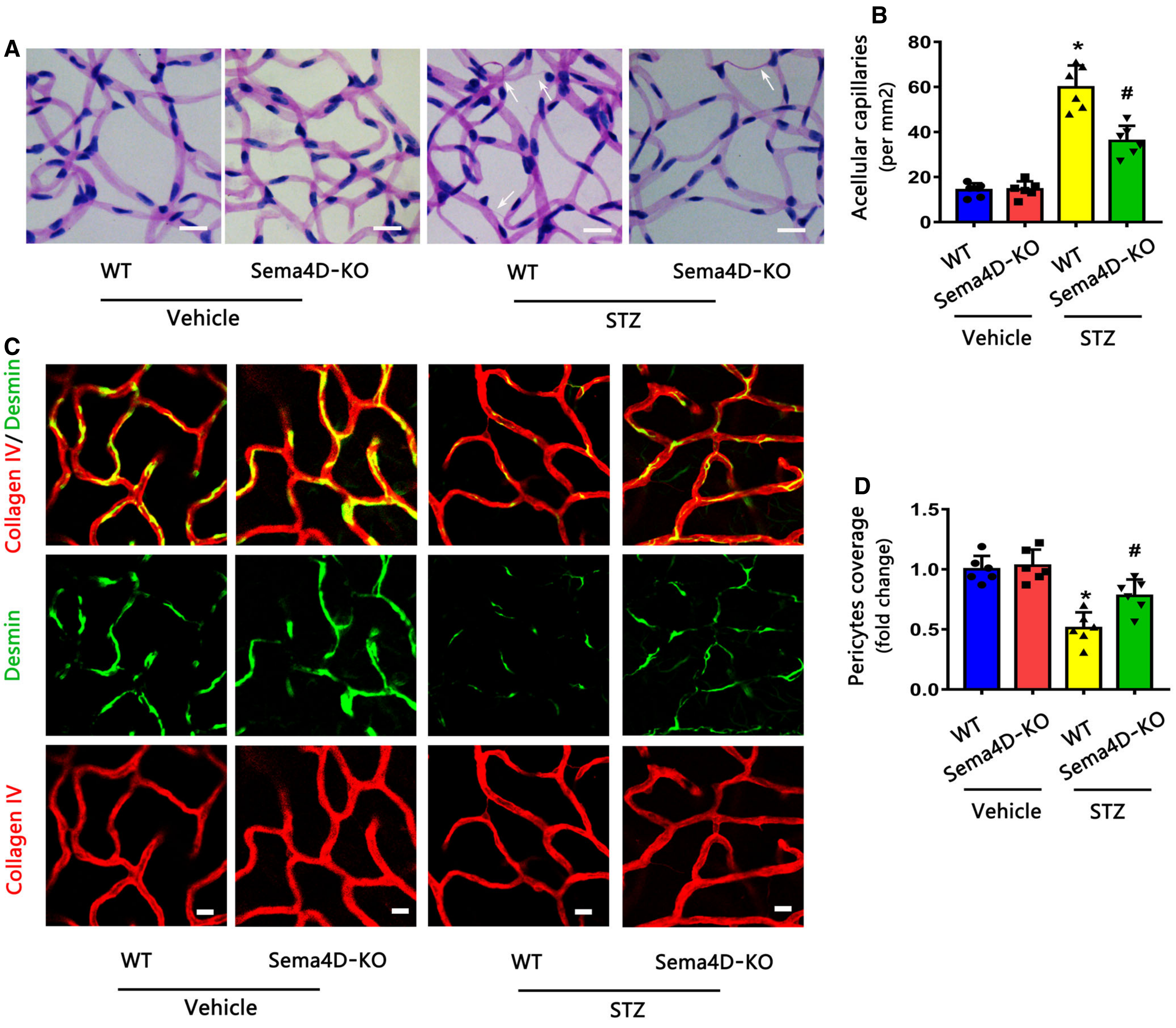

Figure EV1. Sema4D knockout alleviates acellular capillary formation and pericytes loss in STZ model.

A, B Retinal trypsin digestion showed that Sema4D knockout attenuated acellular capillary formation at 3 months in the STZ model (n = 6. Arrows indicate acellular capillaries. Scale bars indicate 20 μm, *P < 0.05 compared with WT + Vehicle group, $^{\#}P$ < 0.05 compared with WT + STZ group).

C, D Immunofluorescence staining of desmin (green, a pericyte marker) with collagen IV (red) at 3 months in the STZ model with or without Sema4D (n = 6, scale bars indicate 10 μm, *P < 0.05 compared with WT + Vehicle group, $^{\#}P$ < 0.05 compared with WT + STZ group).

Data information: Data are means ± SD. Statistical test and P-values are reported in Appendix Table S3.

© 2020 The Authors

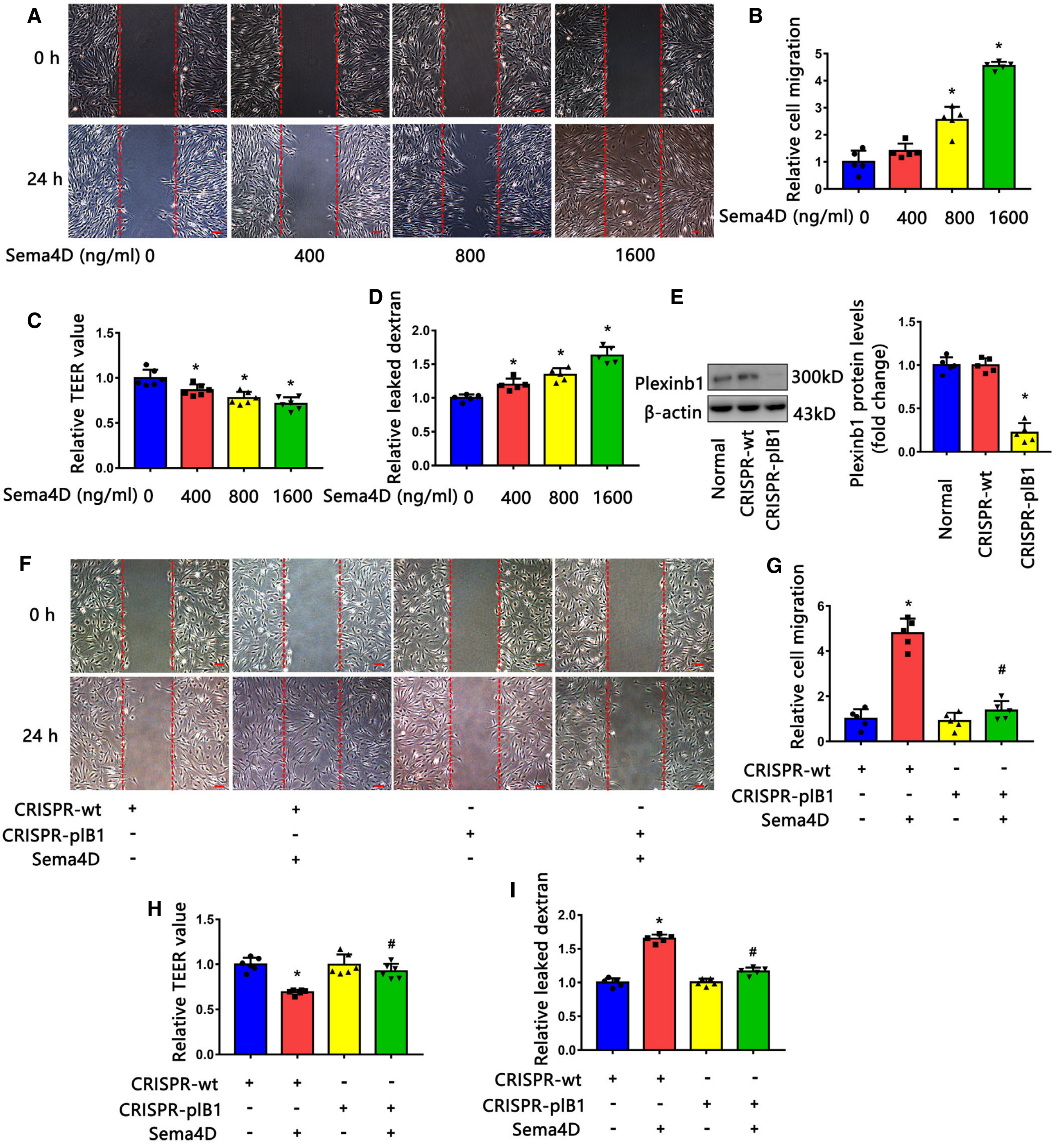
A
0 h
24 h
Sema4D (ng/ml) 0
400
800
1600
B
Relative cell migration
Sema4D (ng/ml) 0 400 800 1600
C
Relative TEER value
Sema4D (ng/ml) 0 400 800 1600
D
Relative leaked dextran
Sema4D (ng/ml) 0 400 800 1600
E
Plexinb1
β-actin
300kD
43kD
Normal
CRISPR-wt
CRISPR-plB1
Plexinb1 protein levels (fold change)
F
0 h
24 h
CRISPR-wt
CRISPR-plB1
Sema4D
G
Relative cell migration
H
Relative TEER value
I
Relative leaked dextran

Figure EV2.

Figure EV2. Sema4D regulates endothelial cell function via the PlexinB1 receptor.

A, B Wound-healing assays indicated that recombinant Sema4D promoted endothelial cell migration in a dose-dependent manner (n = 5. The vertical red lines indicate the border of the wound. Scale bars indicate 100 μm, *P < 0.05 compared with 0 ng/ml Sema4D group).

C, D Trans-endothelial electrical resistance (TEER) values and dextran permeability assays showed that recombinant Sema4D promoted endothelial monolayer leakage in a dose-dependent manner (n = 6 in C, n = 5 in D, *P < 0.05 compared with 0 ng/ml Sema4D group).

E Knockdown efficiency of PlexinB1 in endothelial cells transfected with lentivirus-mediated CRISPR-plB1 (n = 5, *P < 0.05 compared with CRISPR-wt group).

F–I Endothelial cells transfected with lentivirus-mediated CRISPR-wt or CRISPR-plB1 were treated with or without 1600 ng/ml recombinant Sema4D, and then, wound healing (F and G), TEER value (H), and dextran permeability (I) were measured (n = 5 in G, I. n = 6 in H. The vertical red lines indicate the border of the wound in F. Scale bars indicate 100 μm, *P < 0.05 compared with CRISPR-wt group, $^{\#}P$ < 0.05 compared with CRISPR-wt + Sema4D group).

Data information: Data are means ± SD. Statistical test and P-values are reported in Appendix Table S3.
Source data are available online for this figure.

Figure EV3. Sema4D knockout attenuates the PlexinB1 downstream signaling pathways in vivo.

A, B Western blotting was performed to detect the phosphorylation of Src, VE-cadherin, and Fak in whole-mount retinas at P17 in the normal condition or in the OIR model with or without Sema4D (n = 6, *P < 0.05 compared with WT group in normal condition, $^{\#}P$ < 0.05 compared with WT group in OIR).

C, D Immunofluorescence staining of VE-cadherin (green) and collagen IV (red) demonstrated VE-cadherin continuity in retina at 3 months in the STZ model with or without Sema4D (n = 6, scale bars indicate 10 μm, *P < 0.05 compared with WT + Vehicle group, $^{\#}P$ < 0.05 compared with WT + STZ group).

E, F Immunofluorescence staining of N-cadherin (green) and collagen IV (red) demonstrated N-cadherin coverage in retina at 3 months in the STZ model with or without Sema4D (n = 6, scale bars indicate 10 μm, *P < 0.05 compared with WT + Vehicle group, $^{\#}P$ < 0.05 compared with WT + STZ group).

Data information: Data are means ± SD. Statistical test and P-values are reported in Appendix Table S3.
Source data are available online for this figure.

 © 2020 The Authors

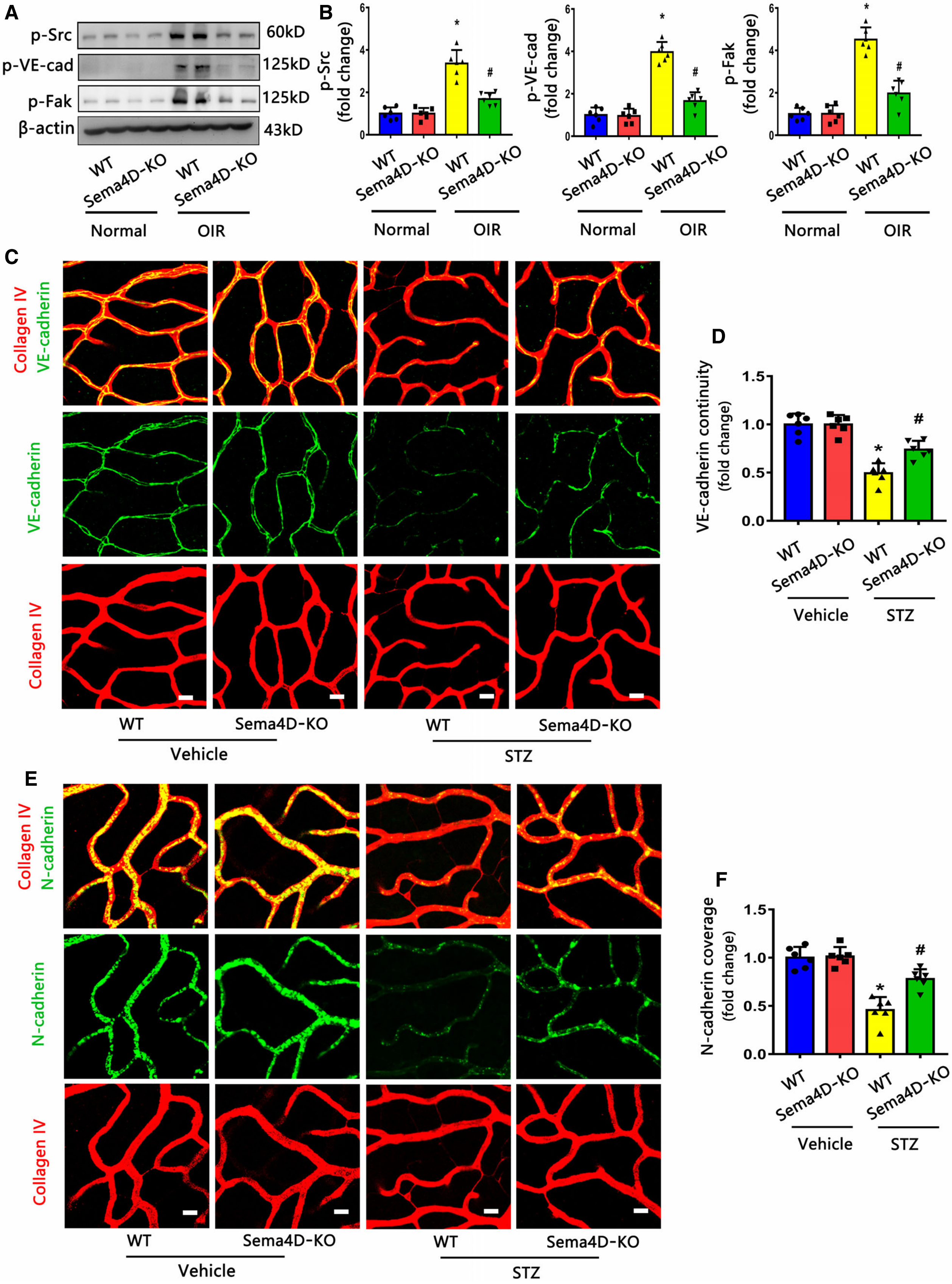
A
p-Src
p-VE-cad
p-Fak
β-actin
60kD
125kD
125kD
43kD
WT
Sema4D-KO
WT
Sema4D-KO
Normal
OIR
B
p-Src (fold change)
p-VE-cad (fold change)
p-Fak (fold change)
Normal
OIR
C
Collagen IV VE-cadherin
VE-cadherin
Collagen IV
WT
Sema4D-KO
Vehicle
STZ
D
VE-cadherin continuity (fold change)
E
Collagen IV N-cadherin
N-cadherin
Collagen IV
F
N-cadherin coverage (fold change)

Figure EV3.
