# Supplementary figures and images for: Inhibition of Sema4D/PlexinB1 signaling alleviates vascular dysfunction in diabetic retinopathy
Source: EMBO Mol Med. 2020 Jan 13;12(2):e10154. doi: 10.15252/emmm.201810154 (PMC7005627; doi:10.15252/emmm.201810154)

Figure EV3A

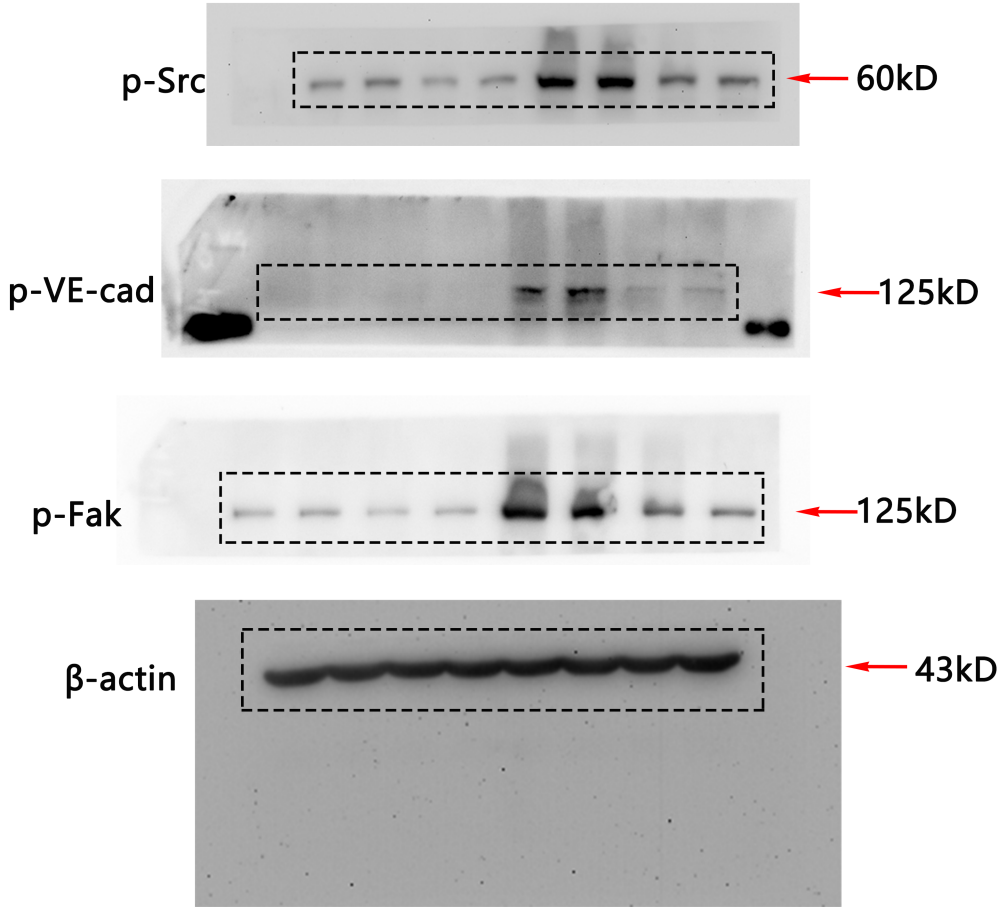

Supplement: Supplementary file 3 — Source Data for Expanded View [file EMMM-12-e10154-s008.zip › EV_source_data/EMM-2018-10154_Source_data_for_FigureEV3.pdf]

Figure EV2E

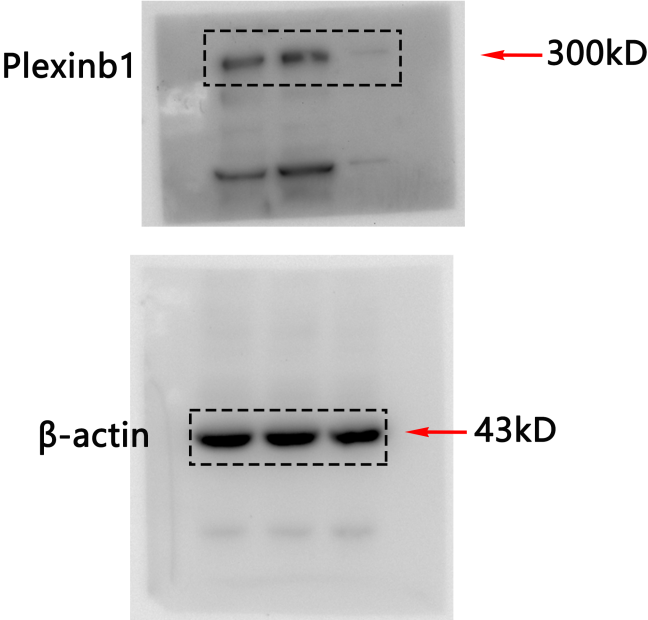

Supplement: Supplementary file 3 — Source Data for Expanded View [file EMMM-12-e10154-s008.zip › EV_source_data/EMM-2018-10154_Source_data_for_FigureEV2.pdf]

Figure 2B

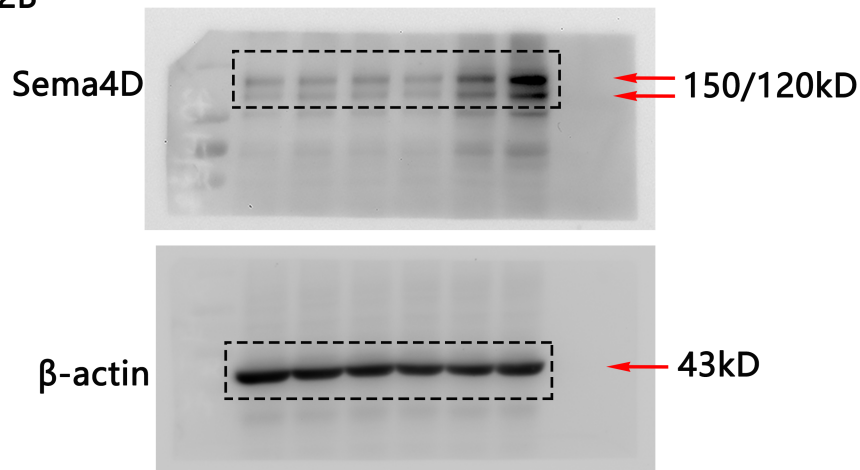

Figure 2D

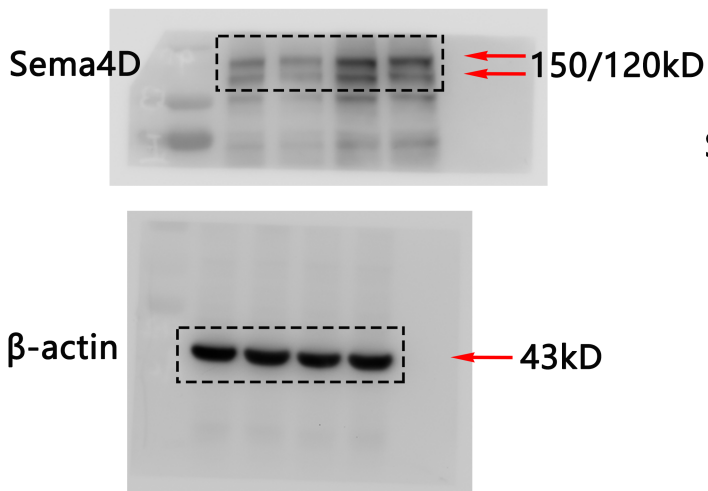

Figure 2F

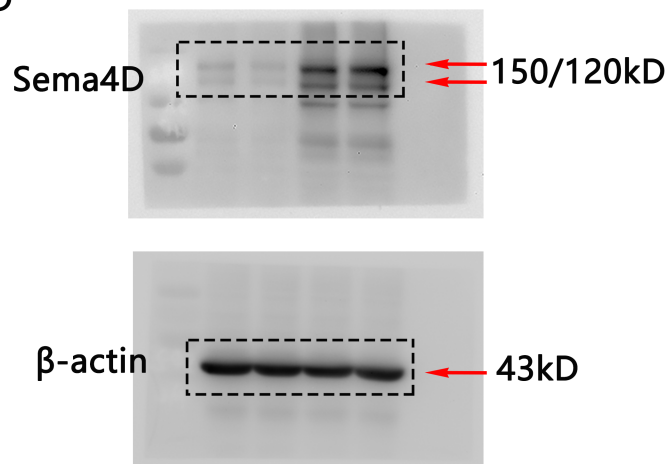

Supplement: Supplementary file 5 — Source Data for Figure 2 [file EMMM-12-e10154-s003.pdf]

**Figure 4A**

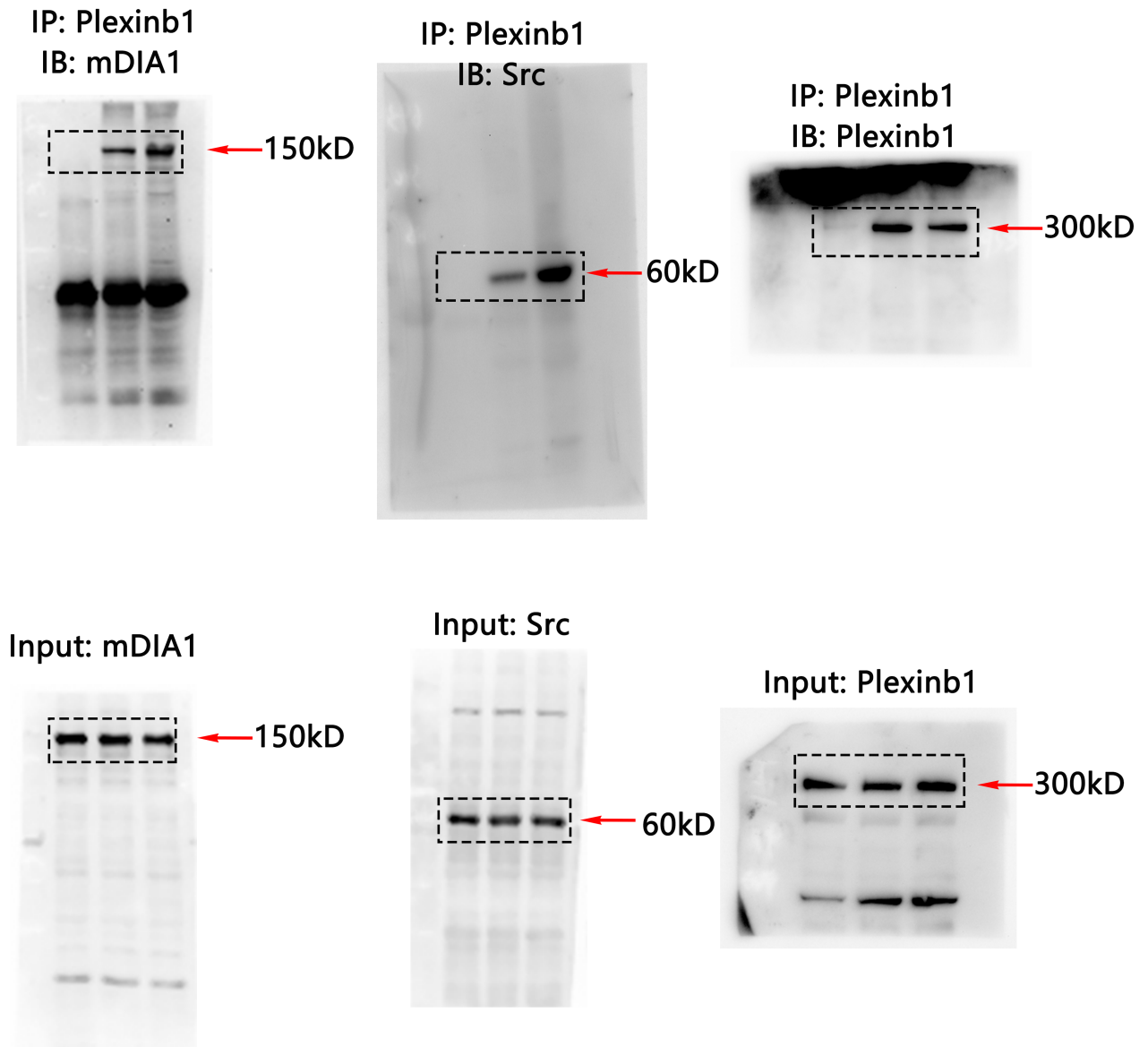

Figure 4B

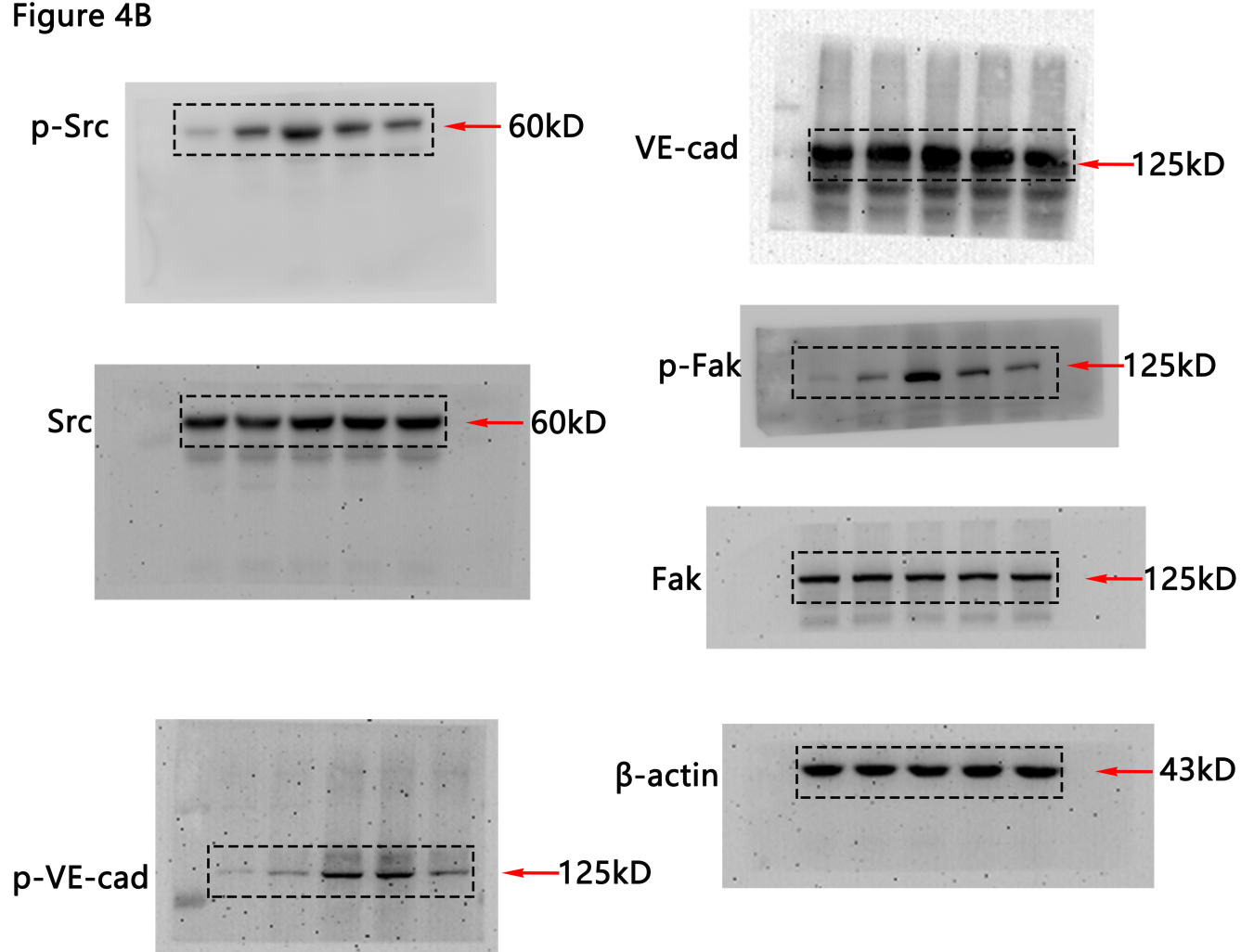

Figure 4D

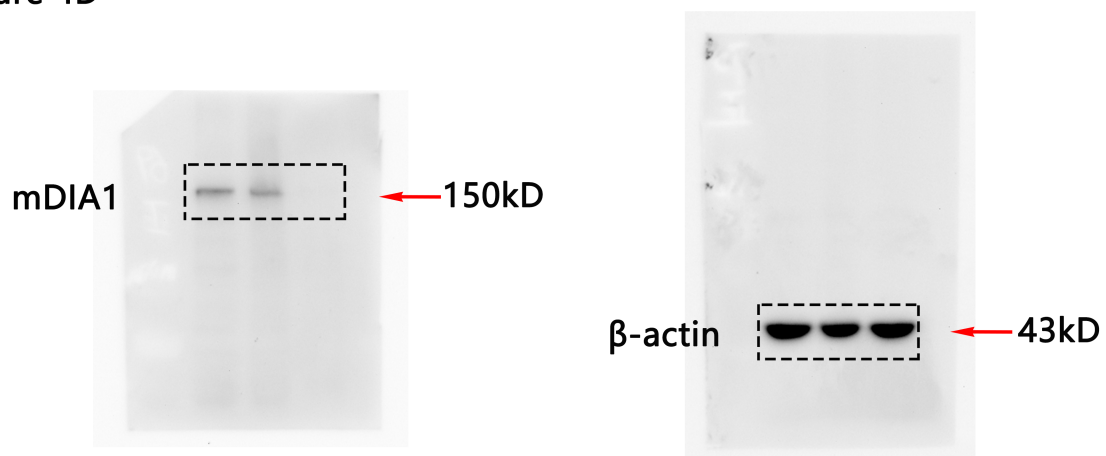

Figure 4E

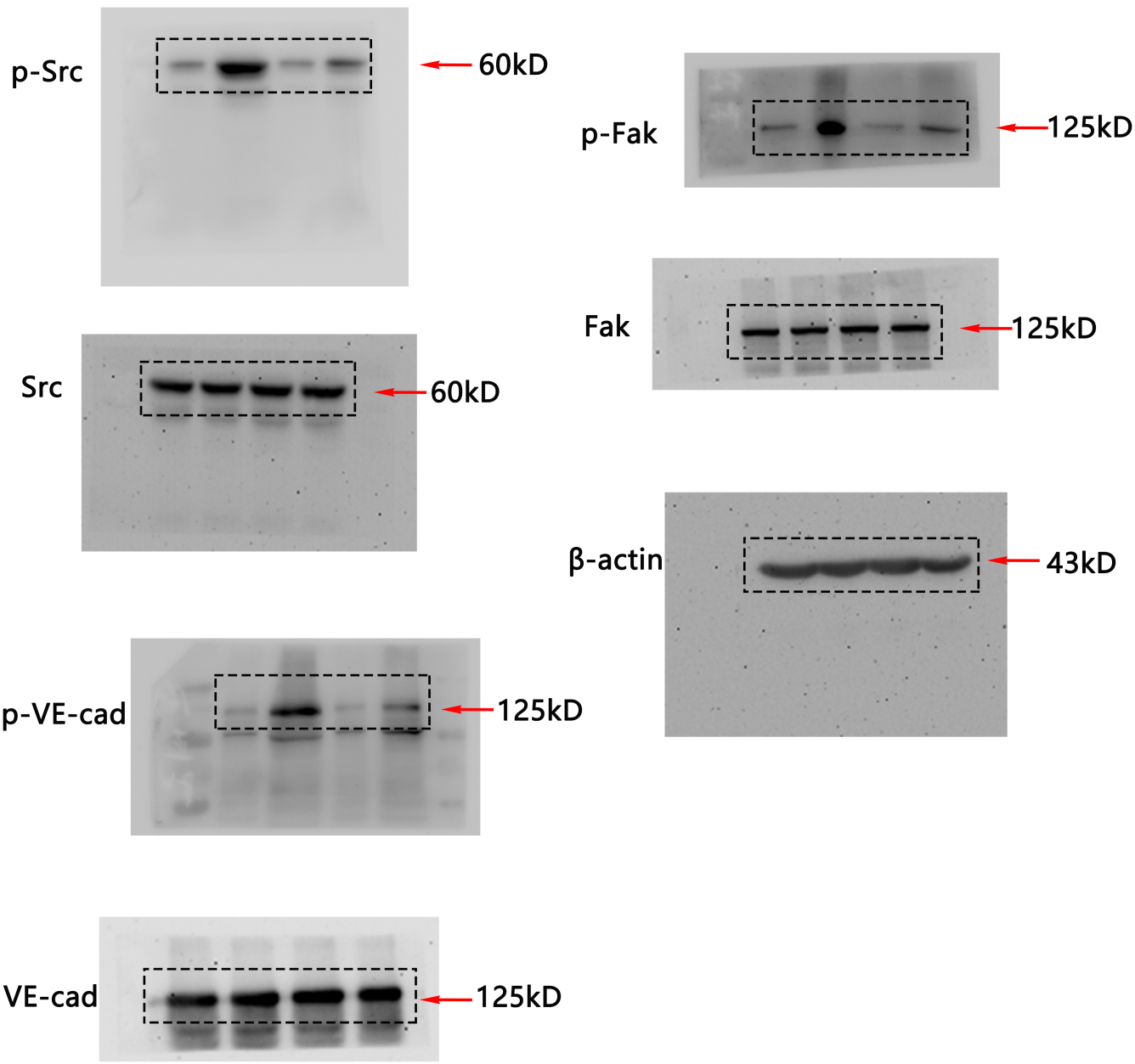

Supplement: Supplementary file 6 — Source Data for Figure 4 [file EMMM-12-e10154-s004.pdf]

Figure 5D

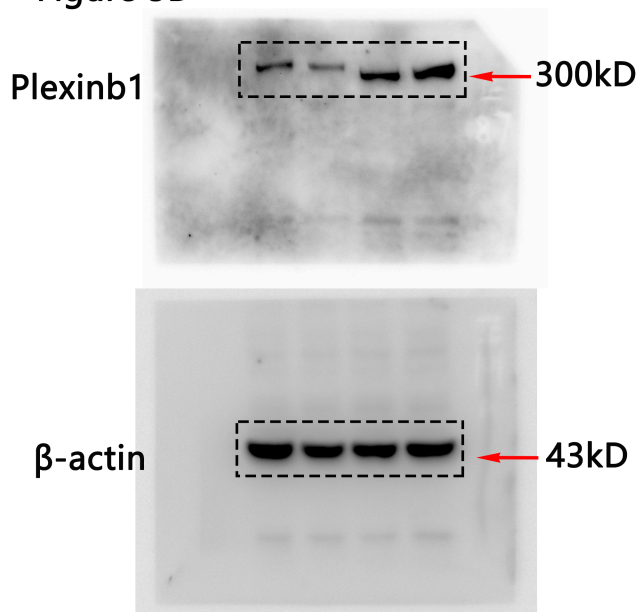

Figure 5F

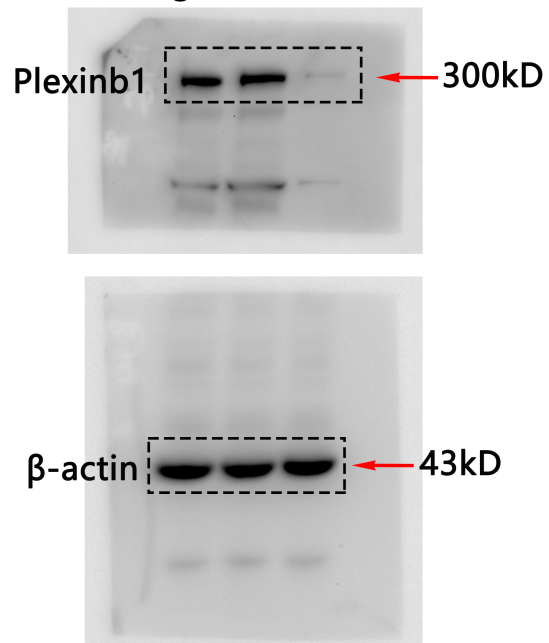

Figure 5L

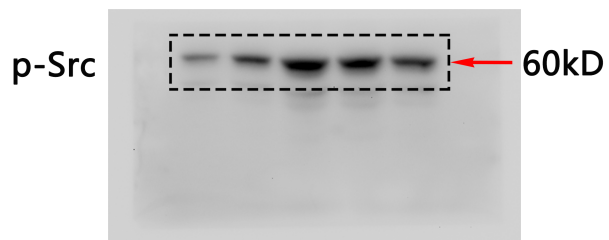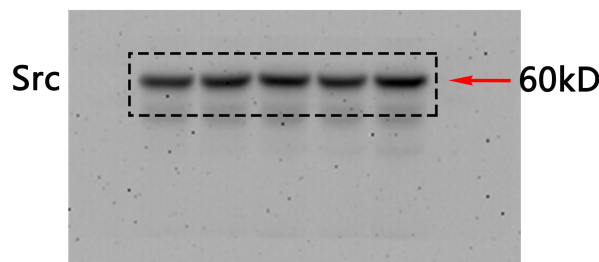

Figure 5N

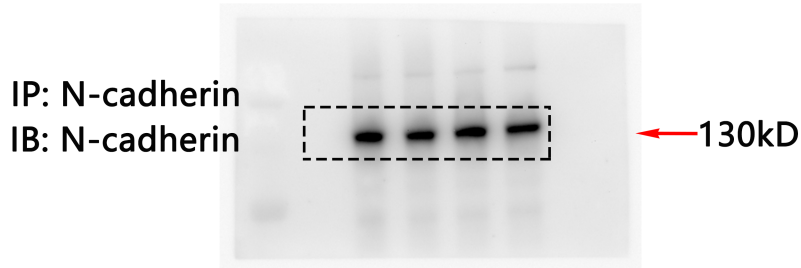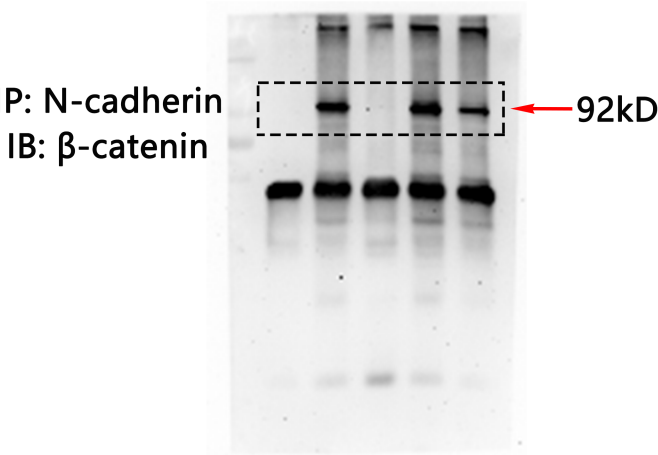

Input: N-cadherin

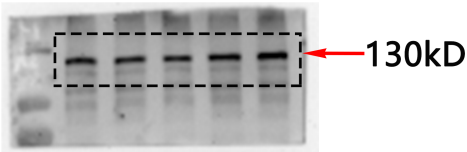

Input:  $\beta$ -catenin

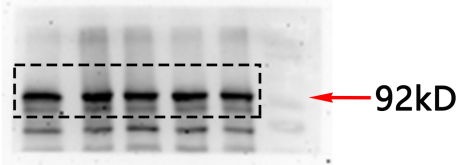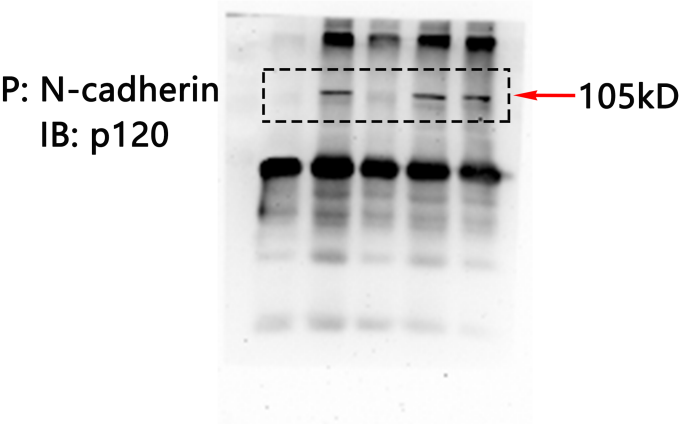

Input: p120

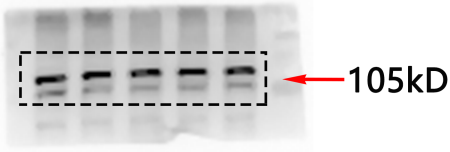

Supplement: Supplementary file 7 — Source Data for Figure 5 [file EMMM-12-e10154-s005.pdf]

Figure 6C

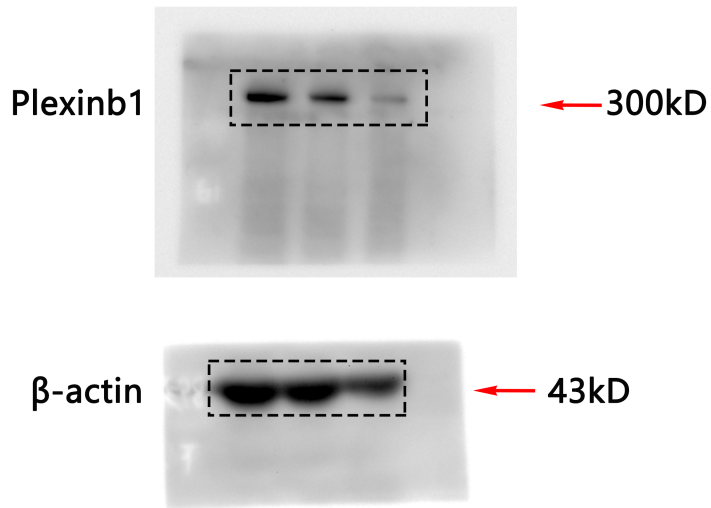

Figure 6J

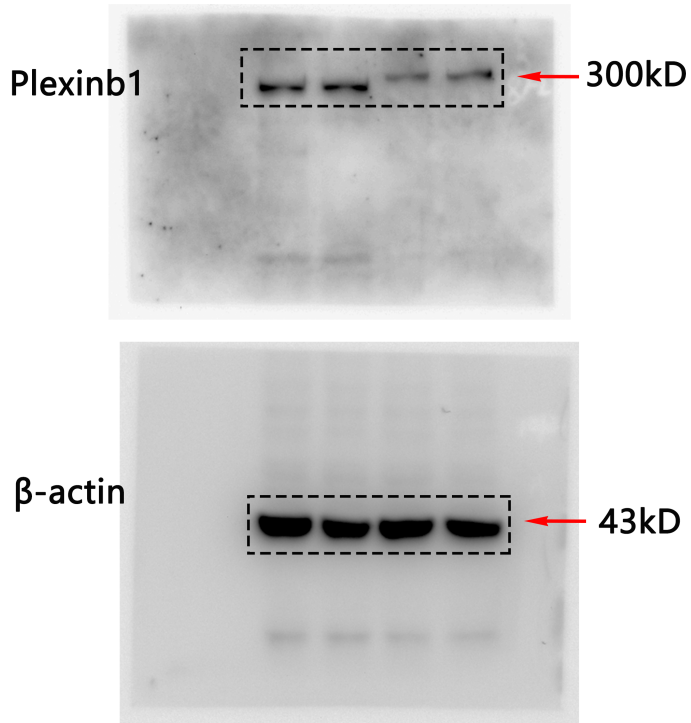

Supplement: Supplementary file 8 — Source Data for Figure 6 [file EMMM-12-e10154-s006.pdf]

Figure7J

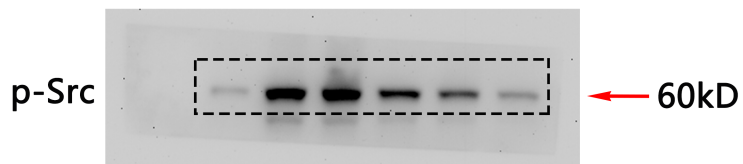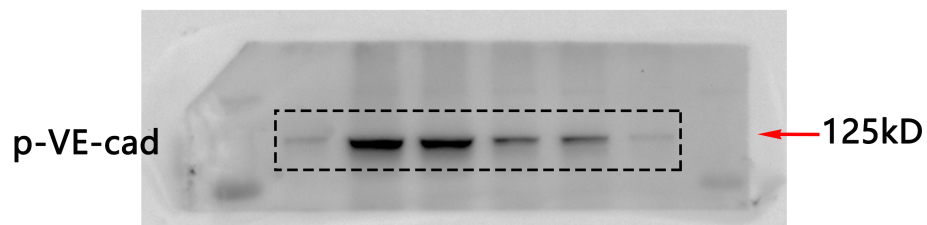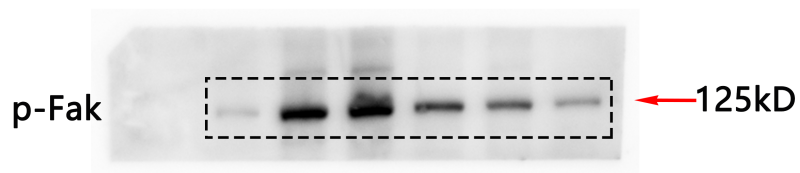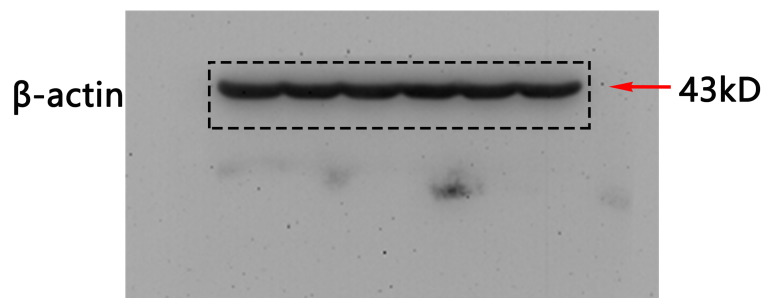

Supplement: Supplementary file 9 — Source Data for Figure 7 [file EMMM-12-e10154-s007.pdf]
